# Mid- and long-term evaluation of an alternative to the Liu' modified Bentall procedure for aortic root aneurysm

**DOI:** 10.3389/fcvm.2025.1647188

**Published:** 2025-11-07

**Authors:** Yong Wang, Weitie Wang, Tonghua Du, Maoxun Huang, Mixia Li, Keyan Liu, Hulin Piao, Zhicheng Zhu, Tiance Wang, Kexiang Liu

**Affiliations:** Department of Cardiovascular Surgery of the Second Hospital of Jilin University, Changchun, China

**Keywords:** aortic root aneurysm, modified bentall technique, mid-term follow-up, Marfan syndrome, aortic dissection

## Abstract

**Background:**

Due to complications such as aortic root bleeding and high coronary tension with the conventional Bentall technique to treat aortic root aneurysms, new methods have been developed to enhance patient outcomes. This study aimed to describe a novel modification of the Bentall procedure for aortic root replacement and to report on its mid- and long-term follow-up outcomes.

**Methods:**

Patients diagnosed with aortic root aneurysm were enrolled in the study. The inclusion criteria included diameter of ascending aorta larger than 50 mm and the aortic valve with organic lesions. Data were collected including surgical time, aortic clamping time, cardiopulmonary bypass time, and pre-discharge computed tomography angiography findings.

**Results:**

Eighty-eight patients (nine with Marfan syndrome), including 69 men (78.4%) and 19 women (21.6%), underwent aortic root replacement using our new root reconstruction technique from 2011 to 2020 at our hospital. The patients’ mean age was 43.4 ± 11.7 years (range, 20–71 years). Of them, 35 (39.8%) had a Stanford type A aortic dissection (dissection group), while 53 (60.2%) had an aortic root aneurysm (aneurysm group). The patients’ in-hospital mortality rate was 2.3% (one case of multiple organ dysfunction syndrome, one case of arrhythmia). The mean aortic cross-clamp and cardiopulmonary bypass times were 120.9 ± 27.1 min and 159.2 ± 37.9 min, respectively. The follow-up rate was 94.2% (81/86) for a mean duration of 55 ± 23 months (range, 6–120 months). Follow-up mortality occurred in three cases (3.7%), including one death due to a traffic accident, one death due to cerebral hemorrhage, and one sudden death of unknown reasons. No patients required an aortic root re-operation during follow-up. The overall survival rate was 98.8%, 95.9%, and 95.9% after 48, 96, and 120 months, respectively.

**Conclusions:**

Our initial experience suggests that this technique is feasible and safe, with promising mid- and long-term outcomes in our series. These descriptive results justify further comparative studies to evaluate its role as a potential alternative for the treatment of aortic root aneurysms.

## Introduction

In 1968, Bentall and De Bono used aortic root composite valve graft replacement to treat an aortic root aneurysm. The original Bentall technique included composite valve graft replacement with coronary ostial reimplantation (1). However, the Bentall technique is associated with proximal hemorrhage, uncontrolled bleeding from coronary artery ostial anastomosis, and pseudoaneurysm formation (2, 3).

The aortic button technique was developed to decrease the coronary tension and bleeding at the reimplantation site; however, it did not improve postoperative aortic root bleeding (4, 5). The Cabrol and modified Cabrol techniques reduce coronary tensions in the suture lines but require a long graft prosthesis, which increases the risk of kinking, angulation, and thrombosis (6–8). The supra-annular aortic wall reinforcing suture (SAWR) technique is easily performed and may improve proximal hemostasis; however, coronary complications persist (9). Thus, inclusion techniques were developed to reduce the “dead” space between the aortic annulus, coronary anastomosis, and sinuses of Valsalva (10). This study aimed to describe the modified inclusion technique and report on its mid- and long-term outcomes.

## Methods

### Patient selection

This retrospective and consecutive series study was approved by the Ethics Board of the Second Hospital of Jilin University (no. 2023-199), which waived the requirement for informed consent for all patients. Between March 2011 and October 2020, 88 consecutive patients were diagnosed with aortic root aneurysm by computed tomography angiography. The inclusion criteria were as follows: (I) diameter of ascending aorta larger than 50 mm, (II) The aortic valve with organic lesions. The exclusion criteria were as follows: (I) contraindications to ceasing preoperative antithrombotic therapy; (II) severe cardiac dysfunction (ejection fraction <35%); and (III) Behcet's disease.

### Surgical procedure

**Figure 1:**
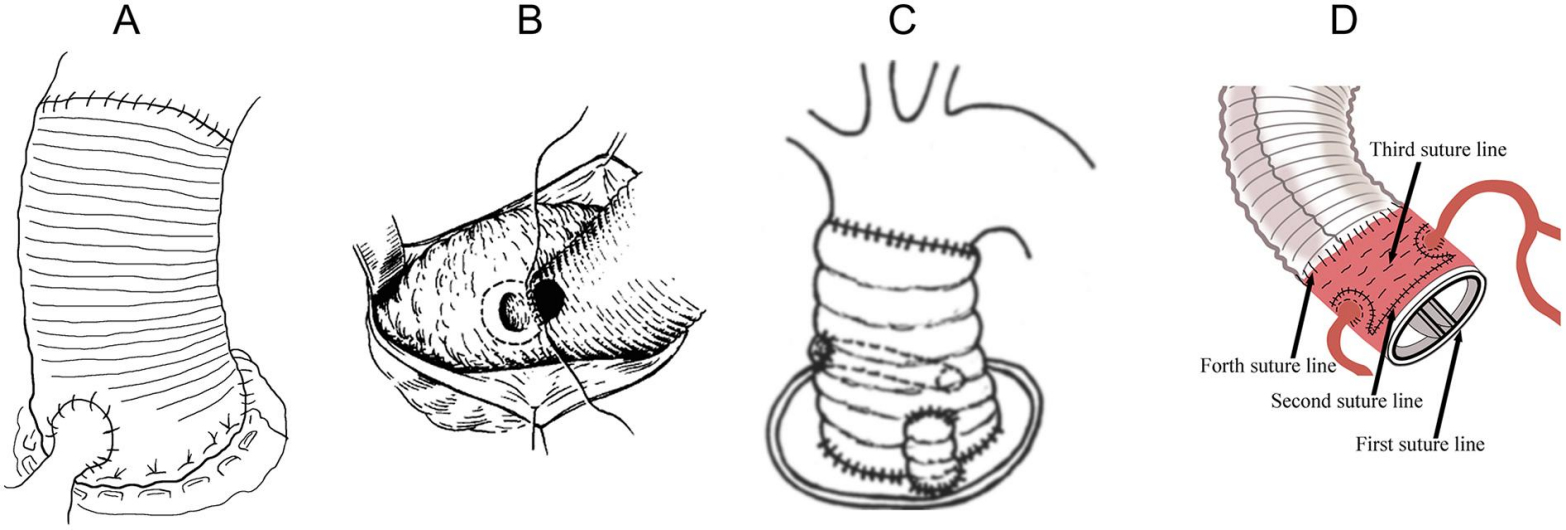
Bentall procedures. **(A)** The classic Bentall. **(B)** The inclusion technique. **(C)** Supra-annular aortic wall reinforcing suture. **(D)** Liu's modified Bentall procedure.

General anesthesia was induced with each patient in the supine position. Through a median sternotomy, cardiopulmonary bypass (CPB) was established via cannulation of the ascending aorta or femoral artery and the superior and inferior vena cava in the aneurysm group. The femoral arteries and the axillary artery were used in the dissection group. The left side of the heart was vented through the right superior pulmonary vein. The ascending aorta was clamped proximal to the innominate artery and transected above the sinotubular junction. Myocardial protection was achieved with antegrade cold-blood cardioplegia or histidine-tryptophan-ketoglutarate solution infused directly through the coronary ostium or via retrograde perfusion through the coronary sinus with topical cooling for myocardial protection. The aortic valve cusps were excised. Annulus size was measured to select the suitable heart valve and vascular prosthesis. A composite graft was prepared by intraoperative suturing of the valves into the vascular grafts and suturing to anastomose the composite graft into the rim of the aortic annulus (first suture line) (Figure 1).

**Figure 2:**
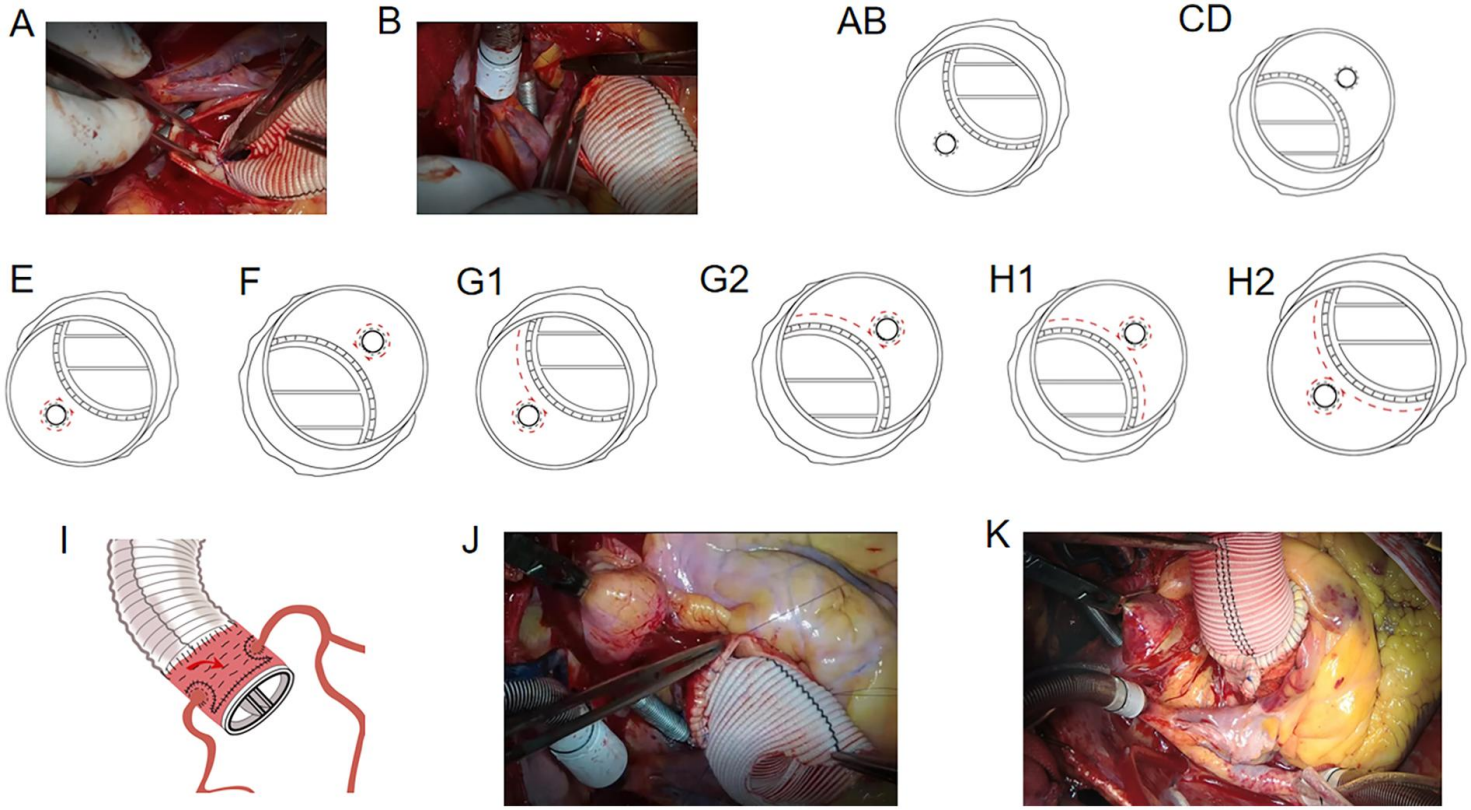
**(A)** suturing of the vascular graft to the lower 1/4–1/3 left coronary ostium. **(B)** Suturing of the other 2/3–3/4 vascular graft to the remaining 2/3–3/4 left coronary ostium. (C, D) Suturing of the right coronary ostium to the vascular graft. **(E)** Suturing from the right lower corner of the left coronary artery to the left lower corner of the left coronary artery. **(F)** Enhancement of the right coronary ostium. **(G1-2)** Horizontal mattress suture above the aortic valve ring from the left corner of the left coronary artery to the right lower corner of the right coronary artery with penetrating suturing. **(H1-2)** Suturing from the left lower corner of the right coronary artery to the right lower corner side of the left coronary artery. **(I)** Horizontal mattress suture between the vascular prosthesis and the aortic wall. **(J)** Suturing of the upper wall of the aortic root with the vascular graft. **(K)** Folding and suturing a part of the aortic wall of the non-coronary aortic sinus.

Circular holes were cut in the aortic prosthesis at the site of the coronary ostia. The lower one-quarter of the vascular graft was sutured to the lower one-quarter of the left coronary ostium as deeply as possible using a 5-0 polypropylene suture string (Figure 2A). We then sutured the other three-quarters of the vascular graft to the remaining three-quarters of the left coronary ostium using the inside-to-out technique, followed by the returning-to-the-inside technique through all layers of the wall of the aortic and vascular graft (Figure 2B). The reconstruction of the left coronary ostium was then completed (Figures 2A,B). The right coronary ostium was sutured to the vascular graft using the same method (Figures 2C,D). The 4-0 polypropylene suture string was used to enhance the coronary ostia with the vascular graft. The suture string was started from the right lower corner of the left coronary artery to the left lower corner of the left coronary artery in a “*Ω*” shape (Figure 2E). The same method was used to enhance the right coronary ostium (Figure 2F). A horizontal mattress suture was placed above the aortic valve ring from the left corner of the left coronary artery to the right lower corner of the right coronary artery with penetrating suturing (Figures 2G1,G2) and from the left lower corner of the right coronary artery to the right lower corner of the left coronary artery (Figures 2H1,H2) (second suture line).

Thereafter, a 4-0 polypropylene horizontal mattress suture was placed between the vascular prosthesis and the aortic wall of the non-coronary aortic sinus or the opposite side using the inside-to-out technique to eliminate the false lumen (Figure 2I) (third suture line). Finally, the upper wall of the aortic root was sutured with the vascular graft (Figure 2J) (fourth suture line). If the diameter of the aortic root was too large, part of the aortic wall of the non-coronary aortic sinus or the opposite side was folded and sutured with the vascular prosthesis to reduce the false lumen (Figure 2K). For patients in the dissection group, a piece of artificial blood vessel was cut and positioned outside the aortic wall for suturing (second and third suture lines) to prevent adventitial bleeding. Next, the anastomosis between the distal ascending aortic vascular and proximal aortic root vascular grafts was completed.

### Data collection and statistical analysis

Continuous data are expressed as mean ± standard deviation, while categorical variables are expressed as number (percentage). The follow-up data were analyzed using the Kaplan–Meier actuarial method to estimate survival probabilities. All statistical analyses were conducted using SPSS 20.0 (SPSS Inc., Chicago, IL, USA).

## Results

The 69 men (78.4%) and 19 women (21.6%) had a mean age of 43.4 ± 11.7 years (range, 20–71 years). Of them, 35 (nine with Marfan syndrome) had a Stanford type A aortic dissection (dissection group) and three had pericardial tamponade. Sixteen patients had mitral regurgitation, six had ischemic heart disease, and 62 had hypertension. The mean aortic root diameter was 66.1 ± 9.65 mm, 66.4 ± 10.3 mm in dissection subgroup and 65.8 ± 9.9 mm in aneurysm subgroup, respectively. The subjects’ preoperative characteristics are presented in Table 1.

**Table 1:** Baseline and procedural characteristics.

Item	Patients	Dissection subgroup	Aneurysm subgroup	P/*X*^2^	t
	(*n* = 88)	(*n* = 35)	(*n* = 53)		
Age (years old)	43.4 ± 11.7	41.8 ± 10.9	45.3 ± 12.3	0.176	1.366
Male	69 (78.4%)	26 (74.3%)	43 (81.1%)	0.445	0.584
Weight (kg)	70.2 ± 13.4	72.9 ± 9.1	69.3 ± 10.8	0.108	1.627
EF (%)	57.2 ± 8.1	57.1 ± 7.6	57.5 ± 8.9	0.218	0.828
Previous cardiac operation	2 (2.3%)	1 (2.86%)	1 (18.9%)	0.765	0.089
Marfan syndrome	9 (10.2%)	5 (14.3%)	4 (7.55%)	0.307	1.043
Pericardial tamponade	3 (3.4%)	1 (2.86%)	2 (3.77%)	0.054	0.817
Mitral regurgitation	16 (18.2%)	4 (11.4%)	12 (22.6%)	0.182	1.782
Ischemic heart disease	6 (6.8%)	1 (2.86%)	5 (9.43%)	0.231	1.435
Diameter of aortic sinus	66.1 ± 9.65	66.4 ± 10.3	65.8 ± 9.9	0.785	0.274
Diameter of ascending artery	47.4 ± 6.8	48.4 ± 6.9	46.8 ± 7.7	0.323	0.994

All surgeries were performed by the same surgeon. All patients underwent aortic root composite valve graft replacement and coronary ostial reimplantation. All procedures were completed with no intraoperative deaths. Overall, the mean aortic cross-clamp time was 120.9 ± 27.1 min, while the mean CPB time was 159.2 ± 37.9 min. By group, the mean aortic cross-clamp time was 105.7 ± 18.5 min for the aneurysm group vs. 143.6 ± 21.1 min for the dissection group, while the mean CPB time was 145.0 ± 38.8 min for the aneurysm group vs. 179.3 ± 25.6 min for the dissection group. The mean circulatory arrest time was 35.8 ± 10.1 min in patients with aortic dissection concomitant with aortic arch replacement. The concomitant procedures included 11 mitral valve plasties, five mitral valve replacements, and six coronary artery bypass grafts.

There were 35 patients with Stanford type A aortic dissection. Of them, 27 underwent Liu's procedure as previously described, including ascending aorta replacement, aortic arch repair, and frozen elephant trunk (11); eight underwent ascending aorta replacement and hemi-arch replacement; and three required extracorporeal membrane oxygenation (ECMO) assistance during the procedure (two with Stanford type A aortic dissection, one with aortic root aneurysm). Table 2 presents the intraoperative data.

**Table 2:** Intraoperative data.

Item	Total	Dissection subgroup	Aneurysm subgroup	*X* ^2^	t
	(*n* = 88)	(*n* = 35)	(*n* = 53)		
Circulatory arrest time(min)	35.8 ± 10.1	35.8 ± 10.1	0	–	–
ACC time (min)	120.9 ± 27.1	143.6 ± 21.1	105.7 ± 18.5	8.892	<0.001
CPB time (min)	159.2 ± 37.9	179.3 ± 25.6	145.0 ± 38.8	4.605	<0.001
Mitral valve plasty	11 (12.50%)	2 (5.71%)	9 (16.98%)	2.447	0.118
Mitral valve replacement	5 (5.68%)	2 (5.71%)	3 (5.66%)	0.0001	0.992
CABG	6 (6.82%)	1 (2.86%)	5 (9.43%)	1.435	0.231
Hemi-arch replacement	8 (9.09%)	8 (9.09%)	0	13.326	0.0003
Total arch replacement + FET	27 (30.68%)	27 (30.68%)	0	58.983	<0.001
Size of the vascular graft	27.4 ± 1.9	26.3 ± 1.5	28.4 ± 1.6	6.176	<0.001
Mechanical valves	81	33 (94.28%)	48 (90.57%)	0.398	0.528
Size of the prosthetic valve	22.0 ± 1.3	21.7 ± 1.1	22.2 ± 1.5	1.693	0.094

The mean mechanical ventilation time was 40.2 ± 45.5 h, while the mean 24 h drainage volume was 388 ± 54.2 mL. By group, the mean mechanical ventilation time was 30.1 ± 37.3 h for the aneurysm group vs. 58.5 ± 52.6 h for the dissection group, while the mean Hospital stay time was 10.5 ± 3.7 days for the aneurysm group vs. 15.6 ± 6.4 days for the dissection group (*P* < 0.05). One patient required an intraoperative aortic balloon pump at 2 days postoperative. Fourteen patients (5 in aneurysm group vs. 9 in dissection group, *P* < 0.05) required continuous renal replacement therapy due to renal insufficiency, and 11 patients presented with an elevated preoperative creatinine level. The in-hospital mortality rate was 2.3% (2/88). One patient with a Stanford type A aortic dissection who required ECMO assistance died of multiple organ dysfunction syndrome on postoperative day 9. One patient with aortic root aneurysm died of arrhythmia on postoperative day 2. Neurological events, including transient consciousness disorder, occurred in three patients; none were permanent. There was no need for re-exploration for surgical bleeding. Thirty-two (36.4%) patients did not require blood products transfusion intra- or postoperatively. Since 2016, our surgical team has applied a strict restrictive protocol for blood product transfusion. Among the 40 patients who underwent the new root reconstruction technique after 2016, 24 (60%) did not need a blood transfusion intra- or postoperatively. The mean intensive care unit stay was 4.1 ± 3.1 days, while the mean hospital stay duration was 13.4 ± 4.5 days. Table 3 presents the patients’ postoperative outcomes.

**Table 3:** Postoperative outcomes.

Item	Total	Dissection subgroup	Aneurysm subgroup	*X* ^2^	t
	(*n* = 88)	(*n* = 35)	(*n* = 53)		
ICU stay (day)	4.1 ± 3.1	4.5 ± 2.7	4.0 ± 3.4	0.731	0.467
Mechanical ventilation time (h)	40.2 ± 45.5	58.5 ± 52.6	30.1 ± 37.3	2.964	0.004
Hospital stay (day)	13.4 ± 4.5	15.6 ± 6.4	10.5 ± 3.7	4.773	<0.001
ECMO support	3 (3.4%)	2 (5.71%)	1 (1.89%)	0.938	0.333
IABP support	1 (1.1%)	0	1 (1.89%)	0.668	0.414
24 h drainage (ml)	388 ± 54.2	423 ± 67.9	355 ± 49.5	5.431	<0.001
Re-sternotomy for bleeding	0	0	0	–	–
Renal dysfunction requiring dialysis	14 (15.9%)	9 (25.7%)	5 (9.43%)	4.176	0.041
Neurological events	3 (3.4%)	3 (8.57%)	0	4.703	0.030
Pericardial tamponade	0	0	0	–	–
No blood transfusion	32 (36.4%)	12 (34.3%)	20 (37.7%)	0.108	0.742
Death	2 (2.3%)	1 (2.86%)	1 (1.89%)	0.089	0.765
Multiple organ failure	1 (1.1%)	1 (2.86%)	0	1.532	0.216
Arrhythmia	1 (1.1%)	0	1 (1.89%)	0.668	0.414

**Figure 3:**
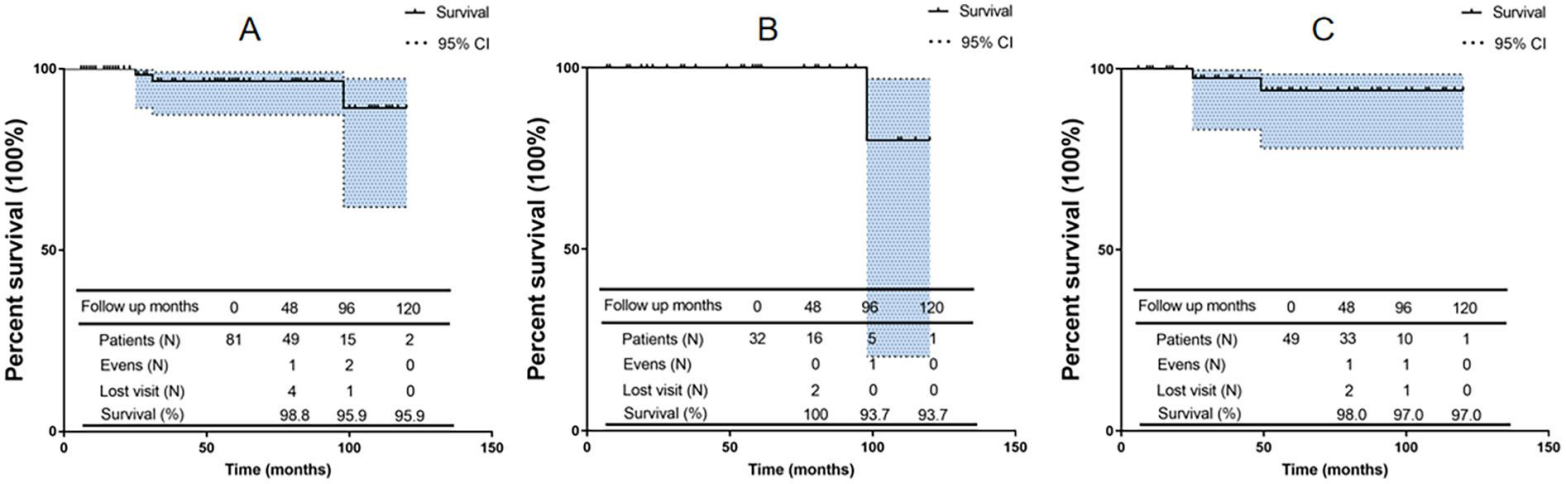
**(A)** the overall survival rate of modified bentall procedure. **(B)** Survival rate of Dissection group. **(C)** Survival rate of of Aneurysm group.

**Figure 4:**
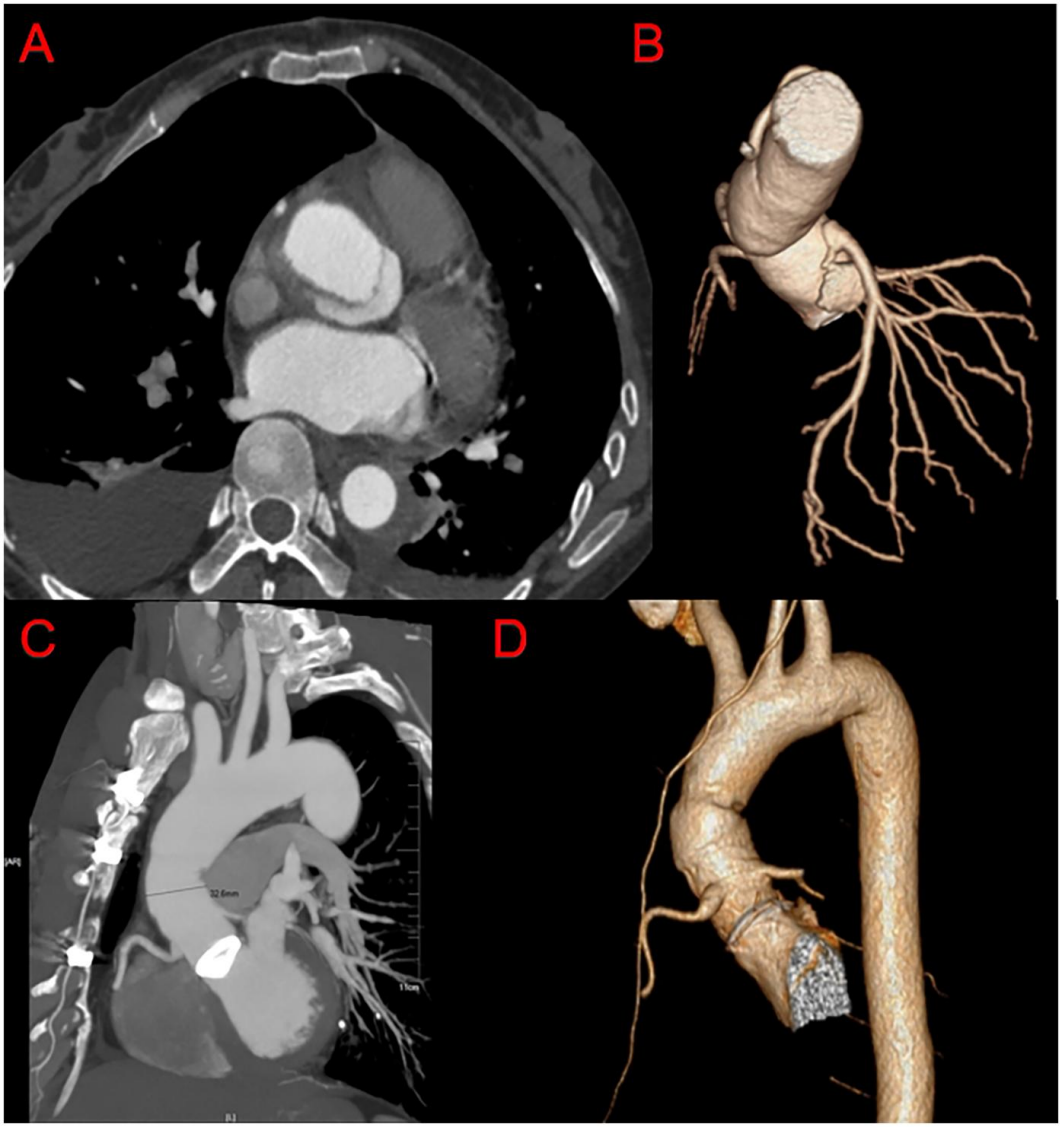
Post-operation CTA imaging of our modified bentall procedure. **(A,B)** Follow up CTA of the patients with the diameter of aortic root enlargement. **(C,D)** Follow up CTA.

The mean postoperative follow-up duration was 55 ± 23 months (range, 6–120 months). Five patients were lost to follow-up; thus, the follow-up rate was 94.2%. Three patients died: one of cerebral hemorrhage (39 months), one in a traffic accident (75 months), and one suddenly (89 months) of unknown reasons. Table 4 presents the follow-up outcomes. The overall survival rate was 98.8%, 95.9%, and 95.9% after 48, 96, and 120 months, respectively (Figure 3A). The survival rate of the dissection group was 100%, 93.7%, and 93.7% after 48, 96, and 120 months, respectively (Figure 3B). The survival rate of the aneurysm group was 98%, 97%, and 97% after 48, 96, and 120 months, respectively (Figure 3C). One patient had aortic root enlargement (Figures 4A,B). The re-examination CTA showed the blood remained between the vascular graft and native ascending aorta. With our third suture line, the blood was limit and did not cause tension. This patient did not receive additional operation. No patients required aortic root re-operation during follow-up (Figures 4C,D).

**Table 4:** Follow up outcomes.

Follow up (55 months)	Follow up	Dissection subgroup	Aneurysm subgroup	*X* ^2^	t
	(*n* = 88)	(*n* = 35)	(*n* = 53)		
Late mortality	3 (3.7%)	2 (6.25%)	1 (2.04%)	0.962	0.327
cerebral hemorrhage	1 (1.2%)	1 (3.13%)	0	1.550	0.213
Traffic accident	1 (1.2%)	0	1 (2.04%)	0.661	0.416
Sudden death	1 (1.2%)	1 (3.13%)	0	1.550	0.213

## Discussion

Surgery of the aortic root for aortic root aneurysms is challenging. Many surgical techniques have been developed and modified in the past five decades. The first standard treatment for aortic root aneurysm was the Bentall procedure, boasting favorable outcomes since 1968 (1). The original Bentall technique included composite valve graft replacement with coronary ostial reimplantation (1). However, the Bentall technique was associated with proximal hemorrhage, uncontrolled bleeding from the coronary artery ostial anastomosis, and pseudoaneurysm formation (2).

Over the years, the Bentall procedure has undergone various modifications to enhance its efficacy, reduce complications, and expand its applicability, including the aortic button technique, Cabrol technique, modified Cabrol technique, SAWR technique, and inclusion technique. The “button technique”, which entails creating islands (or “buttons”) of aortic tissue around the origins of the coronary arteries. These buttons are then sewn into corresponding openings in the graft. This modification aims to reduce the tension on the coronary arteries and improve the patency of the reimplanted vessels, thus minimizing the risk of myocardial ischemia postoperatively. However, This modification did not improve postoperative aortic root bleeding (4). The Cabrol technique and modified Cabrol techniques used an 8- or 10-mm artificial vascular graft instead of direct anastomosis of the coronary arteries, which decreased the coronary tension and reduced the risk of pseudoaneurysm formation (6–8). However, using a long vascular graft prosthesis increases the risk of kinking, angulation, and thrombosis. The SAWR technique was developed and first used in 1998; it can be performed easily and may effectively improve proximal hemostasis, but coronary complications persist (9).

We modified the inclusion technique to reduce coronary artery anastomotic tension and prevent intraoperative aortic root bleeding. Unlike the inclusion technique, our modified method used a SAWR to reduce aortic root bleeding. In our modified technique, only one-quarter of the coronary ostia was anastomosed without penetration sutures. The other three-quarters of the coronary ostia was anastomosed through all the layers of the wall of the aortic and vascular graft, which effectively prevented coronary ostial tearing. The second suture line eliminated the dead space and reduced coronary ostial tension. Therefore, no coronary pseudoaneurysm occurred during follow-up. The third suture line, combined with folding stitching, eliminated the dead space and the tension between the artificial vascular graft and the ascending aorta. An aortic artery-to-right atrial appendage shunt is used in the conventional inclusion technique. However, our method avoided aortic root bleeding, eliminating the need for the aortic artery-to-right atrial appendage shunt. In addition, no re-operations were required to secure bleeding control. Our modified technique achieved satisfactory outcomes.

Among these 88 consecutive patients with aortic root aneurysms, 35 (39.8%) were diagnosed with Stanford type A aortic dissection. The vascular adventitia of the aortic dissection was fragile, and the direct suture vascular adventitia with a vascular graft was prone to bleeding. Therefore, we used a piece of the vascular graft to reinforce the adventitia during aortic root reconstruction and proximal anastomosis and prevent tearing of the vascular adventitia and bleeding. A total of 81 patients were followed up; of them, 50 were followed for 5 years and 17 for 10 years. No pseudoaneurysm was found in patients with a Stanford type A aortic dissection and aortic root aneurysm, and no patients required re-operation.

Prolonged CPB can have potentially detrimental effects on patient outcomes (12). In this study, the CPB and aortic cross-clamp times were slightly (but insignificantly) longer in the dissection vs. aneurysm group. The total CPB (159.2 ± 37.9 min) and aortic cross-clamp (120.9 ± 27.1 min) times were longer than those reported by Roxana et al. (CPB, 112 min; aortic cross-clamp time, 87 min). However, the periprocedural mortality rate was lower in our vs. their study (8%). Although the longer CPB and aortic cross-clamp times, the better outcomes we acquired.

The in-hospital mortality rate of the current study was 2.3%, lower than those reported in the literature (3, 12). Several main factors contributed to the surgical results. First, a high proportion of Stanford type A aortic dissection cases reportedly increases the risk of hospital mortality. Ilker et al. and Pablo et al. reported proportions of Stanford type A aortic dissection of 19.7% and 24.8% and mortality rates of 11.8% and 8.5%, respectively (13, 14). However, 35 (39.8%) patients in our study were diagnosed with a Stanford type A aortic dissection, a higher rate than those of other reports. The follow-up rate was 94.2% (81/86) for 55 months (range, 6–120 months). Second, aortic re-clamping (10.2%) for bleeding reportedly increases the risk of hospital mortality. Mataraci et al. reported that early (30-day) mortality was related to aortic re-clamping (odds ratio, 13.51; 95% confidence interval, 1.18–142.86; *P* = 0.036) (13). In our study, no patient required aortic re-clamping for intraoperative hemostasis due to the application of a piece of artificial blood vessel during distal anastomosis. Mortality occurred in three patients (two in the dissection group, one in the aneurysm group); no patients required aortic root re-operation during follow-up. The overall survival rates were 98.8%, 95.9%, and 95.9% after 48, 96, and 120 months, respectively.

The residual aneurysm of coronary anastomosis after the Bentall procedure is a rare but potentially life-threatening complication, of which computed tomography angiography showed the “goldfish eye” sign upon coronary opening (15, 16). Because of the postoperative arterial wall lesions in Stanford type A aortic dissection or weakness of the congenital aortic wall development in patients with Marfan syndrome, the coronary artery opening transplanted to an artificial vessel may postoperatively form a residual aneurysm resembling a goldfish eye that can lead to sudden death. It is also among the main reasons for re-operation after Bentall surgery. Residual excessive aortic tissue and high tension of coronary ostia may also lead to the goldfish eye sign. In our modified Bentall technique, circular holes were cut smaller in the aortic prosthesis at the site of the coronary ostia, reducing the remaining residual ascending aortic tissue. In addition, the double reinforcement (coronary artery with penetrating suturing and the second suture line) reduced the coronary ostial tension. The goldfish eye sign was not observed in the 35 patients with aortic dissection (including nine with Marfan syndrome).

In this study, the modified Bentall procedure was used only in patients with aortic dissection and aneurysm. The technical principles of our modified procedure—specifically, the multi-layer suturing to eliminate dead space, reduce coronary tension, and achieve secure hemostasis—suggest that it may hold potential for application in other complex root pathologies. Theoretically, it could be suitable for conditions such as prosthetic valve endocarditis destroying the aortic root, intramural hematoma, or acute aortic rupture where secure anastomoses are critical. However, this represents a hypothesis based on our technical outcomes in aneurysms and dissections; future studies are required to formally validate the efficacy and safety of this technique for these expanded indications. We try our best to simplify complex surgical procedures and avoid the complications. Therefore, anyone who can operate the troditional bentall procedure could easily accomplish our modified bentall procedure without learning curve.

This study had some limitations. First, it represents the experience of a single surgeon over a 10-year period, which may limit the generalizability of the findings and introduces the potential for expertise bias. Second, the technique has not yet been validated by other surgeons or centers. Third, the absence of a comparative control group (e.g., patients treated with the conventional Bentall technique) prevents us from drawing any conclusions regarding the comparative efficacy or superiority of this modification. Fourth, the retrospective and observational design inherits its own inherent limitations.

In conclusion, we describe a modified root reconstruction technique that, in our initial experience, was associated with satisfactory descriptive outcomes. While these findings are promising, they are preliminary and require validation through future comparative studies conducted by other surgical teams

## Data Availability

The raw data supporting the conclusions of this article will be made available by the authors, without undue reservation.

## References

[1] Bentall H De Bono A. A technique for complete replacement of the ascending aorta. Thorax. (1968) 23(4):338–9. 10.1136/thx.23.4.338 5664694 PMC471799

[2] Marvasti MA Parker FB Jr Randall PA Witwer GA. Composite graft replacement of the ascending aorta and aortic valve. Late follow-up with intra-arterial digital subtraction angiography. J Thorac Cardiovasc Surg. (1988) 95(5):924–8. 10.1016/S0022-5223(19)35708-3 3283463

[3] Mookhoek A Korteland NM Arabkhani B Di Centa I Lansac E Bekkers JA Bentall procedure: a systematic review and meta-analysis. Ann Thorac Surg. (2016) 101(5):1684–9. 10.1016/j.athoracsur.2015.10.090 26857635

[4] Kouchoukos NT Karp RB Blackstone EH Kirklin JW Pacifico AD Zorn GL Replacement of the ascending aorta and aortic valve with a composite graft. Results in 86 patients. Ann Surg. (1980) 192(3):403–13. 10.1097/00000658-198009000-00016 7416833 PMC1344927

[5] Zhu Y Lingala B Baiocchi M Tao JJ Toro Arana V Khoo JW Type A aortic dissection-experience over 5 decades: JACC historical breakthroughs in perspective. J Am Coll Cardiol. (2020) 76(14):1703–13. 10.1016/j.jacc.2020.07.061 33004136

[6] Cabrol C Pavie A Gandjbakhch I Villemot JP Guiraudon G Laughlin L Complete replacement of the ascending aorta with reimplantation of the coronary arteries: new surgical approach. J Thorac Cardiovasc Surg. (1981) 81(2):309–15. 10.1016/S0022-5223(19)37641-X 7453242

[7] Gök E Başaran M. Long-term outcomes of modified bentall procedure. Koşuyolu Heart J. (2020) 23(2):111–6. 10.5578/khj.69821

[8] Yang S Zhang YY Zi YF Pu L Qian X Ren L Cabrol procedure and its modifications: a systematic review and meta-analysis. J Cardiothorac Surg. (2024) 19(1):153. 10.1186/s13019-024-02642-w 38532449 PMC10964695

[9] Liu KX Yamamoto F Sekine S Goto Y Seki S Yamagishi I An effective method for improving hemostasis in aortic root replacement with a composite graft. Heart Vessels. (1998) 13(5):237–40. 10.1007/BF03257246 10483773

[10] Cebi N Frömke J Walterbusch G. Safe hemostasis by application of a new strict graft inclusion technique for replacement of the aortic root. Ann Thorac Surg. (2003) 76(2):631–2. 10.1016/s0003-4975(03)00026-2 12902129

[11] Liu K Zhu C Zheng X Wang T Xu R Zhu Z A new aortic arch inclusion technique with frozen elephant trunk for type A aortic dissection. Ann Surg. (2020) 271(5):978–83. 10.1097/SLA.0000000000003122 30531532

[12] Al-Mudhaffar SS Alwan A Ouj R Mowaffaq A Kakamad FH Ahmad OF Bentall procedure as a lifesaving surgery: a single center experience. Med Int (Lond). (2023) 3(1):8. 10.3892/mi.2023.68 36733412 PMC9887084

[13] Mataraci I Polat A Kiran B Calişkan A Tuncer A Erentug V Long-term results of aortic root replacement: 15 years’ experience. Ann Thorac Surg. (2009) 87(6):1783–8. 10.1016/j.athoracsur.2009.03.046 19463595

[14] Maureira P Vanhuyse F Martin C Lekehal M Carteaux JP Tran N Modified bentall procedure using two short grafts for coronary reimplantation: long-term results. Ann Thorac Surg. (2012) 93(2):443–9. 10.1016/j.athoracsur.2011.11.003 22269710

[15] Milano AD Pratali S Mecozzi G Boraschi P Braccini G Magagnini E Fate of coronary ostial anastomoses after the modified bentall procedure. Ann Thorac Surg. (2003) 75(6):1797–801. discussion 1802. 10.1016/s0003-4975(03)00015-8 12822618

[16] Xue JR Li B Liu YM Bai T Pan XD Liu NN [Reoperation for residual aneurysm of coronary anastomosis after bentall procedure]. Zhonghua Yi Xue Za Zhi. (2017) 97(20):1589–91. Chinese. 10.3760/cma.j.issn.0376-2491.2017.20.018 28592068

