# Correction: Mid- and long-term evaluation of an alternative to the Liu' modified Bentall procedure for aortic root aneurysm

**DOI:** 10.3389/fcvm.2025.1746856

**Published:** 2026-01-02

**Authors:** Yong Wang, Weitie Wang, Tonghua Du, Maoxun Huang, Mixia Li, Keyan Liu, Hulin Piao, Zhicheng Zhu, Tiance Wang, Kexiang Liu

**Affiliations:** Department of Cardiovascular Surgery of the Second Hospital of Jilin University, Changchun, China

**Keywords:** aortic root aneurysm, modified bentall technique, mid-term follow-up, Marfan syndrome, aortic dissection

The title of this article was erroneously given as “Mid- and long-term evaluation of an alternative to the modified bentall procedure for aortic root aneurysm”. The correct title of the article is “Mid- and long-term evaluation of an alternative to the Liu’ modified Bentall procedure for aortic root aneurysm”.

The original version of this article has been updated.

