# Supplementary material for: Mid- and long-term evaluation of an alternative to the Liu' modified Bentall procedure for aortic root aneurysm
Source: Front Cardiovasc Med. 2025 Nov 7;12:1647188. doi: 10.3389/fcvm.2025.1647188 (PMC12635848; doi:10.3389/fcvm.2025.1647188)
Supplement: Supplementary file 1 [file Table1.pdf]

Supplementary Table 1

Dissection	(n=1144)	Surgical
Dissection (type A)	678 (59.3%)	New aortic root reinforcement+Ascending aorta replacement+FET
Dissection (type B)	466 (40.7%)	TEVAR
Dissection+Mitral valve regurgitation	15 (1.3%)	New aortic root reinforcement+Ascending aorta replacement+FET+MVR/MVP
Dissection+Aortic valve regurgitation	22 (1.9%)	New aortic root reinforcement+Ascending aorta replacement+FET+AVR/AVP
Dissection+Ischemic heart disease	11 (0.9%)	New aortic root reinforcement+Ascending aorta replacement+FET+CABG
Dissection+Root aneurysm	88 (7.7%)	Modified Bentall+Ascending aorta replacement+FET
Dissection+Atrial septal defect	8 (0.7%)	New aortic root reinforcement+Ascending aorta replacement+FET+Repair of atrial septal defect
